# Round Window Stimulation of the Cochlea

**DOI:** 10.3389/fneur.2021.777010

**Published:** 2021-12-14

**Authors:** Herman A. Jenkins, Nathaniel Greene, Daniel J. Tollin

**Affiliations:** ^1^Department of Otolaryngology-Head and Neck Surgery, University of Colorado School of Medicine, Aurora, CO, United States; ^2^Department of Physiology and Biophysics, University of Colorado School of Medicine, Aurora, CO, United States

**Keywords:** round window stimulation, active middle ear prosthesis, electro-mechanical stimulation, hearing loss, cochlear micromechanical response to stimulation

## Abstract

Mixed hearing loss associated with a sensorineural component and an impaired conductive mechanism for sound from the external ear canal to the cochlea represents a challenge for rehabilitation using either surgery or traditional hearing amplification. Direct stimulations of the ossicular chain and the round window (RW) membrane have allowed an improved hearing in this population. The authors review the developments in basic and clinical research that have allowed the exploration of new routes for inner ear stimulation. Similar changes occur in the electrophysiological measures in response to auditory stimulation through the traditional route and direct mechanical stimulation of the RW. The latter has proven to be very effective as a means of hearing rehabilitation in a group of patients with significant difficulties with hearing and communication.

## Introduction

Hearing loss is a common malady that affects people of all ages. Throughout the last century, many different interventions have successfully improved communication skills for deaf and hard-of-hearing individuals. Methods employed have varied, depending on the etiology, degree of hearing loss, and the status of the middle ear. Mild-to-moderate sensorineural hearing loss is easily treated with traditional hearing aids, while cochlear implantation may be required in cases of severe-to-profound loss. A purely conductive hearing loss is often managed with middle ear operative procedures, amplification, or a combination of the two. However, the mixed conductive and sensorineural hearing loss remain a difficult condition to ameliorate. Traditional amplification is often stretched to its limits, having to overcome both a conductive and a sensorineural loss with outcomes often distorted and unappreciated by the patient. Hearing physician-scientists have turned to alternative pathways of stimulation, most commonly osseointegrated bone stimulators (1). In the presence of a significant sensorineural component, direct bone stimulation has proved unsatisfactory, and attention turned to direct round window (RW) membrane stimulation as a possible acceptable alternative.

## Early Studies of the RW

The contribution of the RW to hearing was first suggested in the late mid-17th century with early clinicians proposing that it was a possible route for transmission of vibratory energy to the inner ear (2, 3). The argument as to whether the RW membrane acts as a transmitter or a compensatory egress for vibratory sounds persisted well into the early 20th century, and the need for more scientific knowledge was acknowledged. Kobrak (4) in studying the RW membrane demonstrated that a similar magnitude of vibrational sound conducted through either the eardrum or the RW membrane resulted in equivalent vibrations of the incus, though different in phase (4). He additionally noted that the “tensor reflex” in the opposite ear of the experimental animal during testing was similar for both stimulation sites.

In early patient observations (5), it was observed that dampening the RW in hard-of-hearing patients has improved hearing. Wever and Lawrence (6) on revisiting this study, instead, demonstrated that dampening the RW membrane in cats resulted in a 5–10 dB decrease in the promontory measured cochlear potentials (6). They presumed this to be either the RW input to the cochlear stimulation, as suggested by DuVernay (2) centuries earlier or disruption of inner ear mechanics through added pressure. These authors (7) in a later study noted that a balanced stimulus delivered simultaneously to the oval and RWs resulted in a cochlear potential response that varied with the difference in phase angle of stimulations; a maximal response being measurable when the phases showed the greatest differences and the responses lessened with decreases in this maximal phase angle relationship. In a further study, these authors (8) compared alternative mechanisms of stimulating the cochlea, such as the normal conductive pathway, the RW, and an independent probe inserted through a small apical fenestra of the cochlea with measurements of the cochlear potentials near the RW membrane. They concluded that “the basal and the apical stimuli affect the same regions of the basilar membrane and in essentially the same manner.”

## Early Clinical Intervention in RW Stimulation

With the dissatisfaction of the standard hearing aid and the low acceptance rate in many deafened individuals, interests turned to devise potential alternative methods to stimulate the inner ear. During the latter decades of the 20th century, multiple investigators began to stimulate the ossicular chain directly with mechanically translating devices to produce ossicular chain movement. The devices used different technologies to stimulate the inner ear, such as piezoelectric crystals (9, 10), electromagnetic (11), and electromechanical transducers (12–14), all primarily focusing on ossicular chain translation. At approximately the same timeframe, based on earlier observations (8), investigators used an electromagnetic stimulator (15) and a piezoelectric vibrator (16) to stimulate the auditory system *via* the RW membrane.

Recorded cortical-evoked potentials were similar in response to a broadband click in the external auditory canal and a vibrational stimulation of the RW membrane. The latter group noted that sound stimulation of the ear was more effective than direct mechanical RW stimulation in producing cortical responses.

After multiple attempts to re-establish the traditional ossicular route of stimulation of the inner ear due to disease, further attempts are often doomed to failure. The work of the above investigators (6–8) led Garcia-Ibanez (17) to propose a new method of stimulation of the cochlea, which he termed “Sonoinversion” (17). By isolating the stapes and oval window surgically from the RW, he was able to stimulate the exteriorized RW membrane using both free field sound and, in some cases, a columellar strut. His improved hearing results were similar to those previously demonstrated with the lateral semicircular canal fenestration procedure, an intentional third window into the ear for treating hearing loss in otosclerosis (18).

In such a group of patients who have previously undergone multiple surgeries, Colletti et al. (19) removed the crimping hinge from a Floating Mass Transducer (FMT) (11) and embedded it in the RW niche against the RW membrane (19). The majority of their seven initial patients achieved aided thresholds of 30 dB HL and 100% intelligibility at conversational levels. The authors noted that this was a significant improvement over achievable traditional amplification, due to the inability to deliver a conducted stimulus to the footplate efficiently. These results spawned multiple publications addressing patient results and complications from this new procedure, the vibroplasty.

The vibroplasty has met skepticism by others (20), complaining of movement of the device with time and questionable coupling. Investigators stressed that ideal coupling could be achieved using interposed tissue between the FMT and RW with verification intraoperatively with auditory-evoked response testing, such as electrocochleography (21) and steady state measures (22), with a follow-up radiographic CT imaging (23) to improve clinical results, though a recent publication continues to deny the need (24). A European multicenter study (25) in 2010 noted the improvement in hearing performance in 12 patients, including audiometric measures, sound field thresholds and speech in quiet and noise, and subjective benefits documented by the Abbreviated Profile of Hearing Aid Benefit. A systematic review of the literature (26) using the FMT in conductive hearing loss reviewed 19 studies that met their inclusion criteria. Being still early in the use of the device, they concluded that vibroplasty was beneficial in a speech in quiet, patients-reported outcomes measures, and residual hearing safety. The reviewed existent evidence was moderate-to-low in confidence for determining benefits, due to the variability of test materials, surgical techniques, and lack of any standard methods of comparisons across the studies in preoperative and follow-up testing. A third consensus statement (27) reviewing the state of knowledge for vibroplasty concluded that the operative procedure had become established as a reliable technique in conductive or mixed hearing loss treatment. The authors suggested techniques in the procedure for improving results included wide access exposing the RW membrane, perpendicular placement of the FMT avoiding bony contact, and four-point fixation of the device to maintain proper alignment long term.

Skarzinski et al. (28) in a long-term follow-up of their vibroplasty cases contended that interposing tissue between the RW and the FMT was not necessary if the lips of the RW niche were drilled out (28). However, a later study from this group (29) demonstrated that better stimulation was achieved with interposed tissue. Other authors (30) reported that an overall revision rate of 15.6% was needed to achieve improved performance in vibroplasty, the highest rate found in patients with direct coupling to the RW membrane without interposed tissue. Stable performance was present for up to 7 years post-implantation in series. Recently, investigators confirmed the improvements in all subscales of the Abbreviated Profile of Hearing Aid Benefit (APHAB) in a multicenter Korean study (31), continuing to validate the efficacy and safety of the procedure.

## Research Studies of RW Stimulation

Reviewing the early investigative works (8) and the success of the clinical intervention (19), even in the face of skepticism of many, renewed interests were kindled to better understand the actual stimulation and surgical parameters that contributed to the success and failure of the vibroplasty procedure. These studies relied on the observation that the cochlea could be stimulated by mechanical movement of the RW membrane and the resultant response parameters appeared qualitatively similar, regardless of the route of stimulus delivery (8). This work was further supported by Voss et al. (32), who observed that the cochlea responds essentially to the difference in pressure at the oval and RWs with the response of the basilar membrane being proportional to this difference (32). Presentation of sound to the round and oval windows in the presence of an uncoupled ossicular chain produced results essentially of the same magnitude.

Rosowski et al. (33) outlined testing methods for quantifying the output for implantable middle ear hearing devices by integrating these prior observations (33). Further investigations (34) using the Carina (Otologics Corporation) electromechanical transducer applied this smaller, more defined tip to explore the parameters of reverse stimulation in the live Chinchilla lanigera preparation, comparing the cochlear microphonics (CMs) and stapes velocity in response to acoustical stimulation of the ear and mechanical stimulation of the RW membrane.

**Figure 1:**
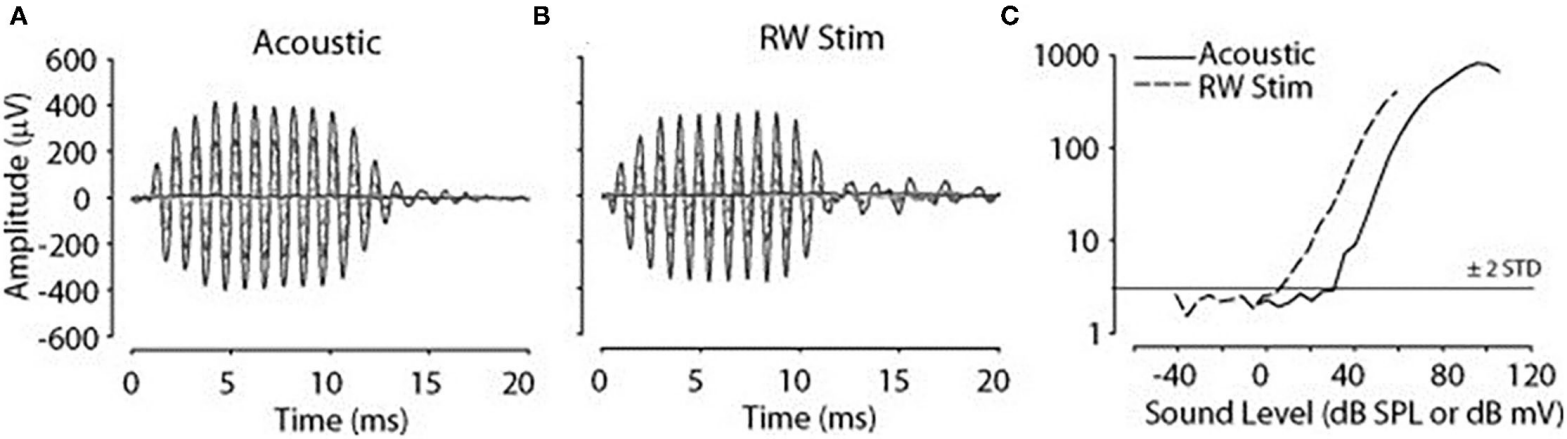
Cochlear microphonic (CM) in response to 1 kHz stimulus. **(A)** Acoustic 10–80 SPL, **(B)** round window (RW) −10 to 60 dB mV, **(C)** CM amplitude vs. stimulus level function, horizontal line threshold of response, dashed line RW stimulation, solid line acoustic.

**Figure 2:**
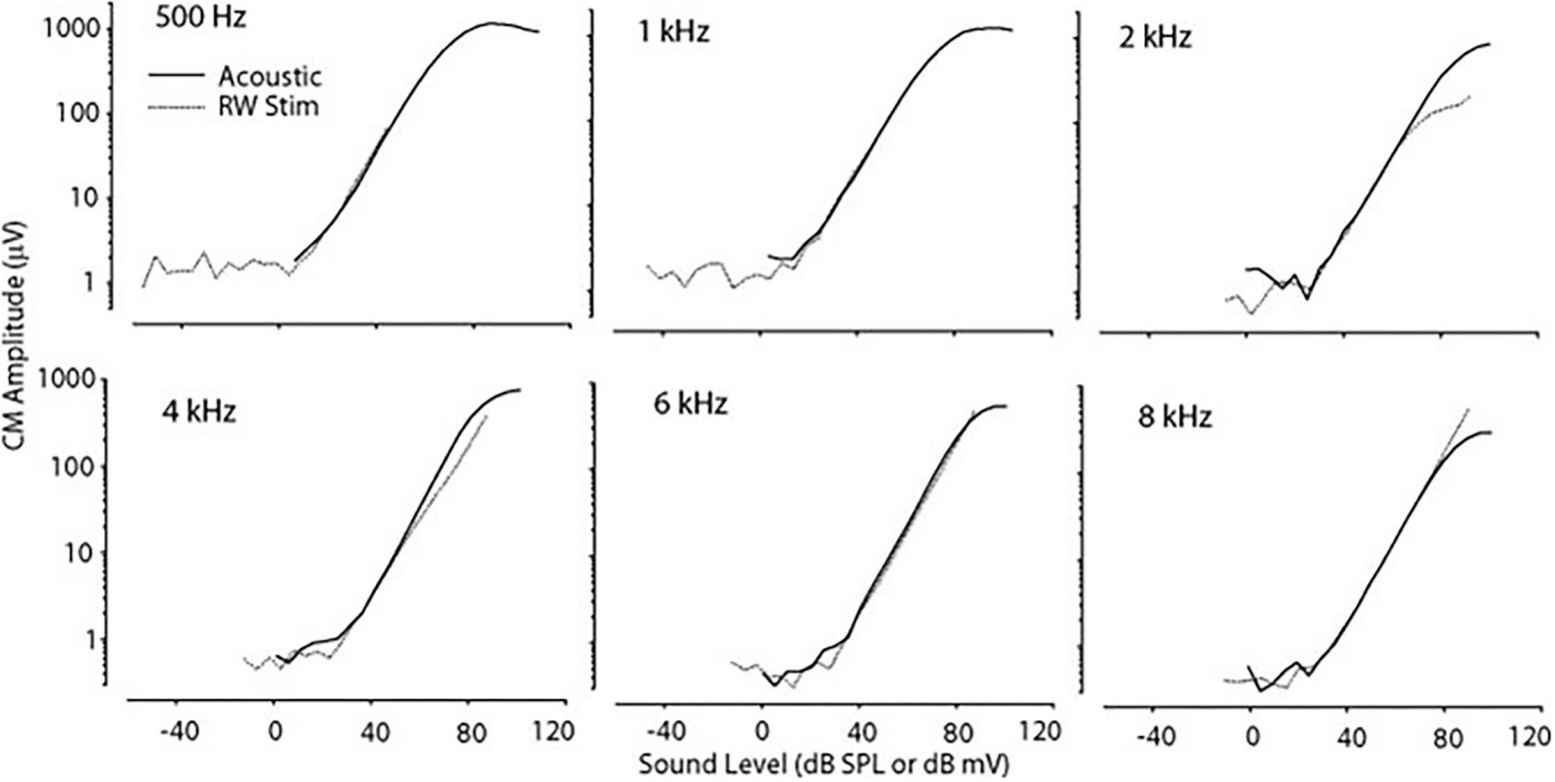
CM vs. stimulus intensity for acoustic and RW stimulation at differing frequencies. The function curve has been moved along the abscissa to remove the effect of delay in the transmitted acoustic stimulus. CM, cochlear microphonic; RW, round window.

**Figure 3:**
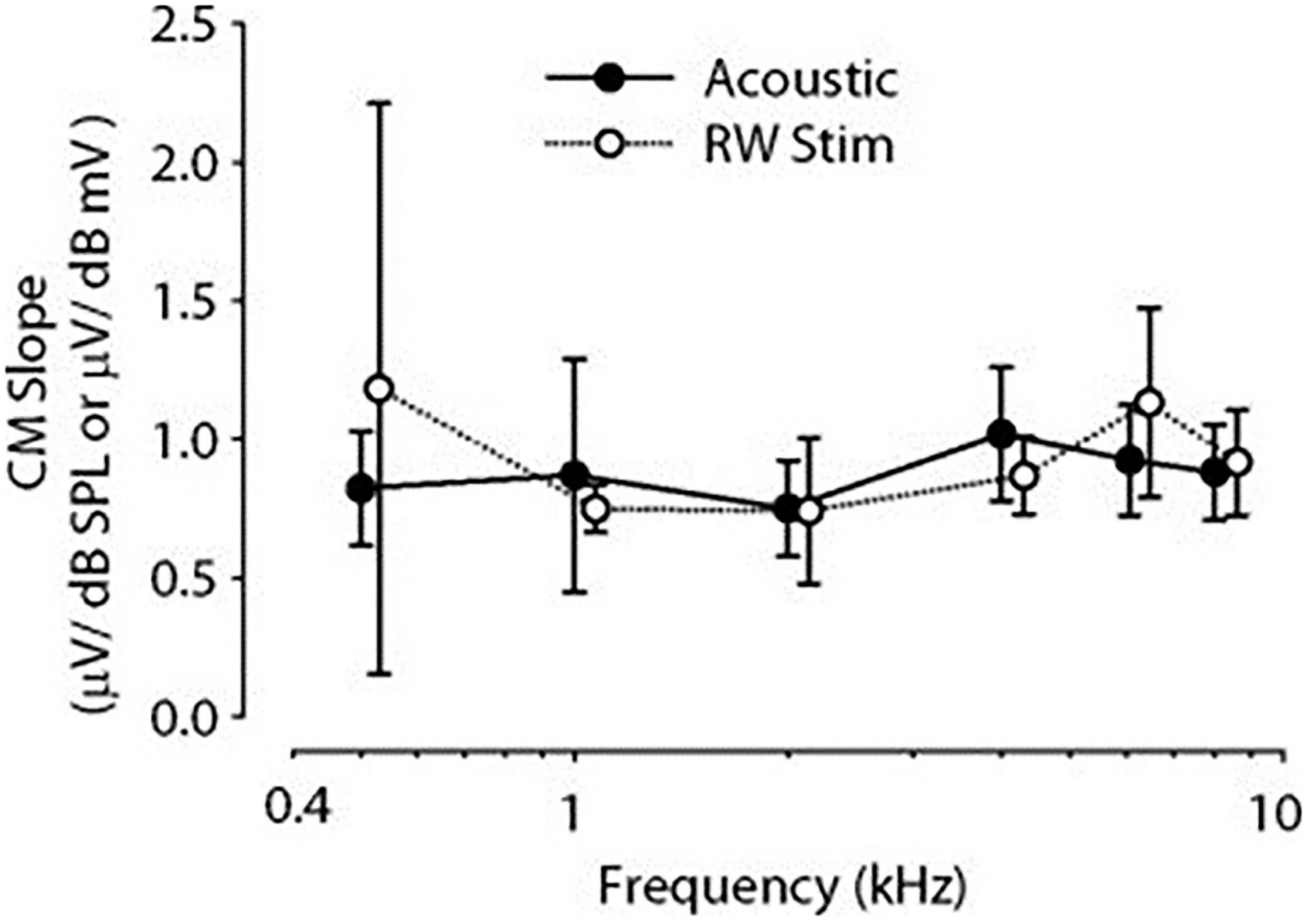
Comparison of CM mean slopes for different frequency stimuli. Acoustic filled circle, RW open circles, error bars ±1 SD. CM, cochlear microphonic; RW, round window.

Cochlear microphonic represents primarily the outer hair cell response of the cochlea, allowing an estimate of the stimulus energy received. CM waveforms were recorded in response to the two different stimulation modes in this live model, and representative waveforms are shown in Figure 1. The CM amplitudes were increased with stimulus intensity and thresholds were decreased with increasing frequency, similarly in both acoustic and electromechanical stimulation with the ossicular chain both intact and disarticulated. After shifting recorded responses to equilibrate the acoustical and mechanical stimulation (33), the functions related to stimulus levels and CM amplitude show nearly identical growth and are independent of frequency over the range 0.5–8 kHz, with no statistical differences observed, as shown in Figure 2. Acoustic and mechanical stimulations result in CM with nearly identical sensitivity and dynamic range profiles, indicating oval and RW stimulation as comparable, as shown in Figure 3.

Ossicular chain disruption, a common scenario, improved CM for frequencies near 3 kHz, however, the differences were relatively minor and were not statistically different. Electro-vibrational transfer functions, defined as stapes velocity normalized to the input signal to the transducer as a function of frequency, were similarly calculated during RW stimulation. With an intact ossicular chain, the peak velocity occurred at 0.68 mm/s/V at 2.8–3 kHz and fell off quickly above and below this peak. With chain disruption, the peak velocity at 2.8–3 kHz was reduced by −6.3 dB to 0.33 mm/s/V, but velocities were nearly doubled across the rest of the range of frequencies tested after disarticulation of the chain, being significant in all animals. Stenfelt et al. (35) had previously demonstrated that motion of the RW membrane at low frequencies reflects stapes motion, where the volume of fluid displaced at the oval window approximately equals that of the RW (35).

Vibroplasty is subject to potential compromise based on surgical techniques applied (27). The potential physiological effects of various implantation parameters involved in the coupling of an active middle ear implant to the RW were further studied in the live Chinchilla lanigera preparation (36). The parameters of loading pressure interposed connective tissue, and angle of stimulation of the RW membrane were evaluated, measuring CM and stapes velocity in response to sinusoidal stimuli generated by an electromagnetic transducer. CMs were measured by an electrode fixed at the RW niche and stapes velocity by laser Doppler velocimetry. The diameter of the tip may be less of a concern in using interposed fascia between the RW membrane and the stimulator tip, potentially decreasing the interference of surrounding bone with a relatively compressible tissue. Overall, repeated loadings and angle of RW stimulation showed only minimal differences in the two measured responses. The interposition of tissue between the stimulator and the RW membrane improved energy transfer (7.6–21.5 dB) between the transducer and cochlea (36).

**Figure 4:**
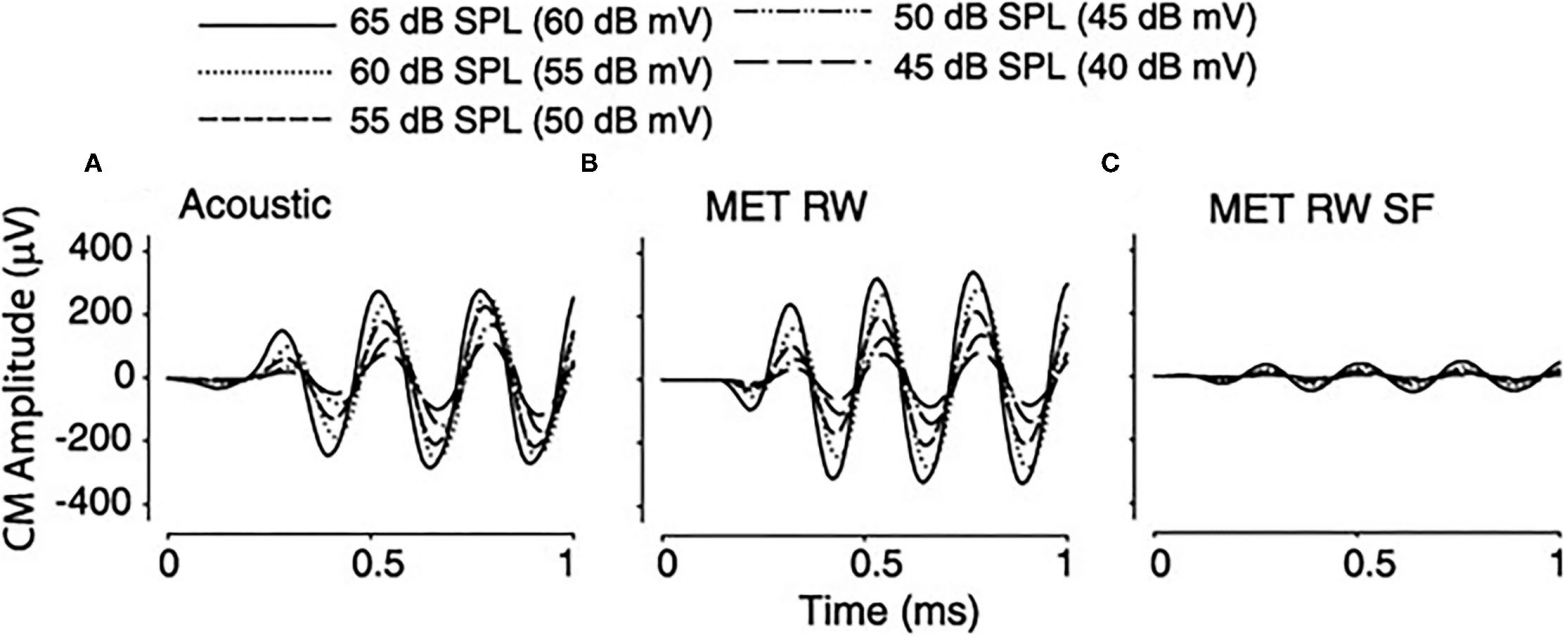
Cochlear microphonic (CM) tracing in a single subject in response to a 2-KHz stimulus with varying stimuli under differing conditions. **(A)** Acoustic 65–45 dB SPL, **(B)** RW 60–40 dB mV, **(C)** round window (RW) with stapes fixation (SF) 60–40 dB mV.

**Figure 5:**
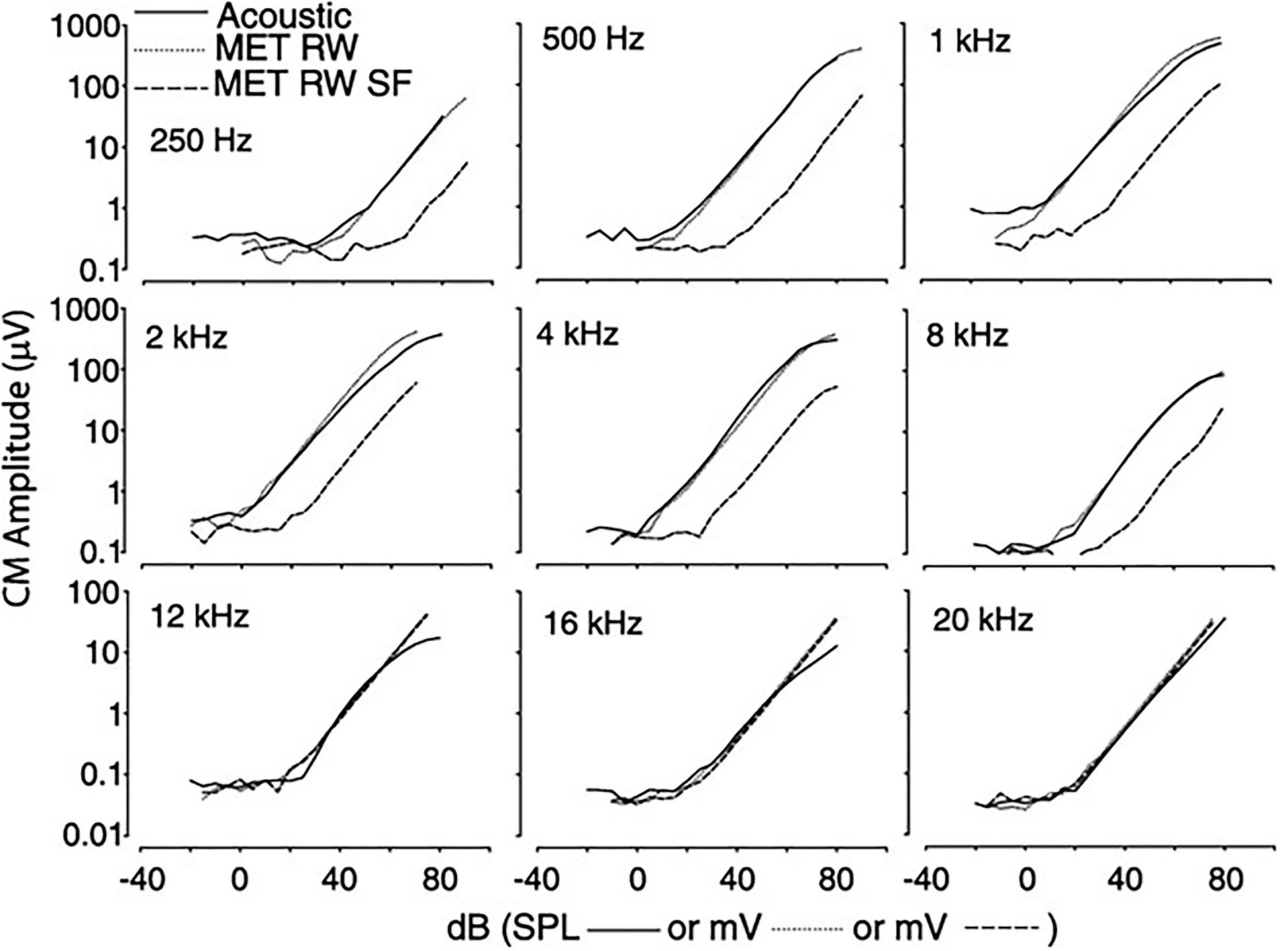
The magnitude of CM vs. stimulus intensity for acoustic (solid line), RW (dotted line), and RW with SF (dashed line). Functions for RW stimulation shifted to align CM thresholds. CM, cochlear microphonic; RW, round window; SF, stapes fixation.

Clinically, fixation of the stapes footplate or RW closure occurs with a high frequency in chronic ear disease. The effect of fixation of the oval and RW on the cochlear response was measured in the adult fat sand rat (37). RW fixation increased air conduction thresholds for clicks on average from 36.4 ± 0.9 to 69.3 ± 4.1 dB Sound Pressure Level (SPL) and fixing the oval window added another 20 dB. Bone conduction was unaffected in both conditions, implying the possible presence of a third window effect. Other authors (38), using the Chinchilla langeria model described above (34), studied the effect of acoustic and RW stimulation in the presence of experimentally induced, Laser Doppler Vibrometer (LDV) confirmed, stapes fixation (SF). Similar waveform morphologies of the CM, compound action potential (CAP), and auditory brainstem responses (ABR) are seen in all experiments, as shown in Figure 4. The morphology of the CM measured at the RW niche was preserved across all stimulus conditions, but SF resulted in attenuation of the amplitude of the waveform for equivalent magnitudes of input stimuli. Similarly, the CM waveform was decreased in amplitude as stimulus intensity decreased. A shorter latency was noted with the transducer-evoked response, attributed to the lack of conductive delay through the ossicular chain. After adjusting for differences in stimulation between decibel sound pressure level and decibel millivolt (33), the response waveforms were essentially identical and independent of the stimulation method. Fixation, as would be predicted, increased the threshold of response. Compared to RW stimulation with normal bony chain, SF significantly increased CM thresholds by a frequency-dependent threshold of 4–13 dB, with lower frequencies showing greater magnitudes of change, compared to higher frequencies, as shown in Figure 5.

**Figure 6:**
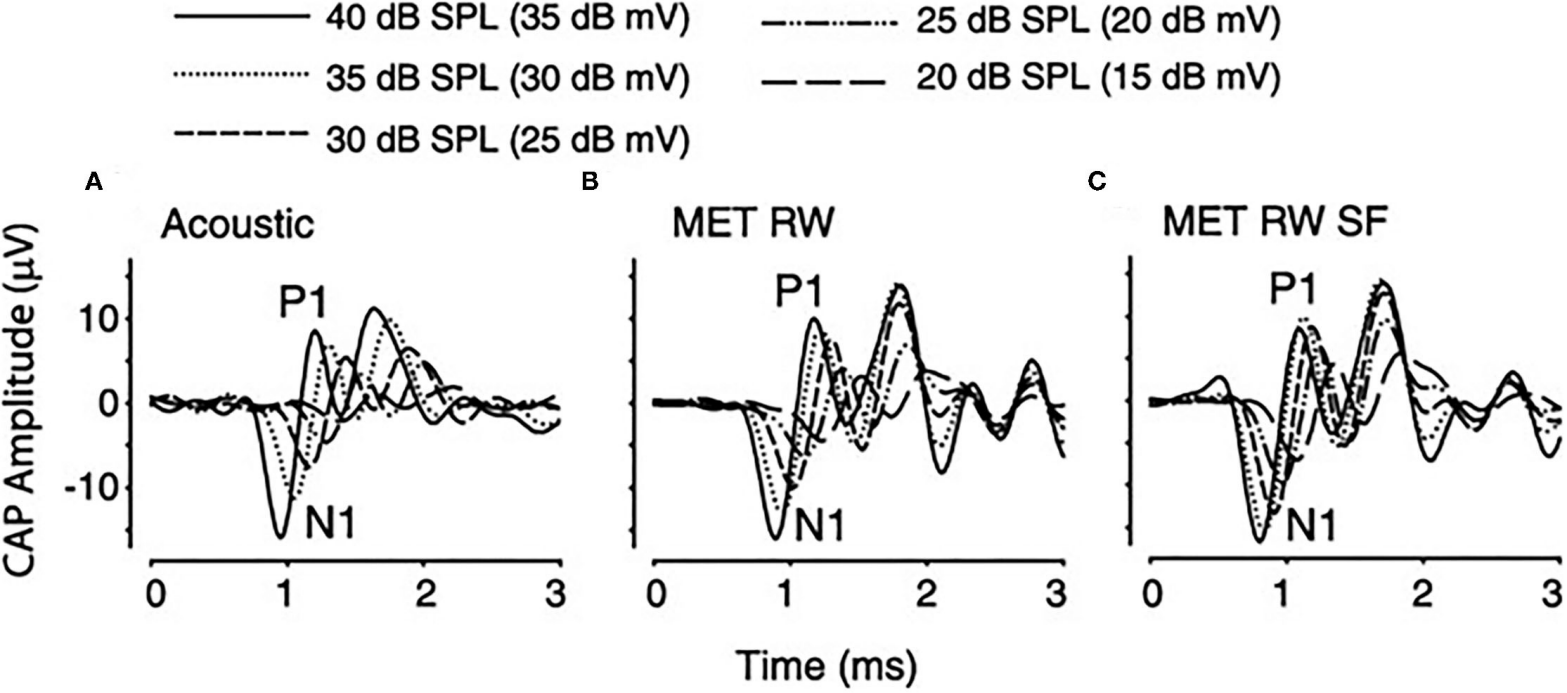
Compound Action Potential (CAP) from one subject using a 4-KHz stimulus during acoustic, RW, and RW with SF stimulation conditions. SF, stapes fixation; RW, round window; MET, middle ear transducer; SPL, sound pressure level, N1-P1, action potential wave form.

**Figure 7:**
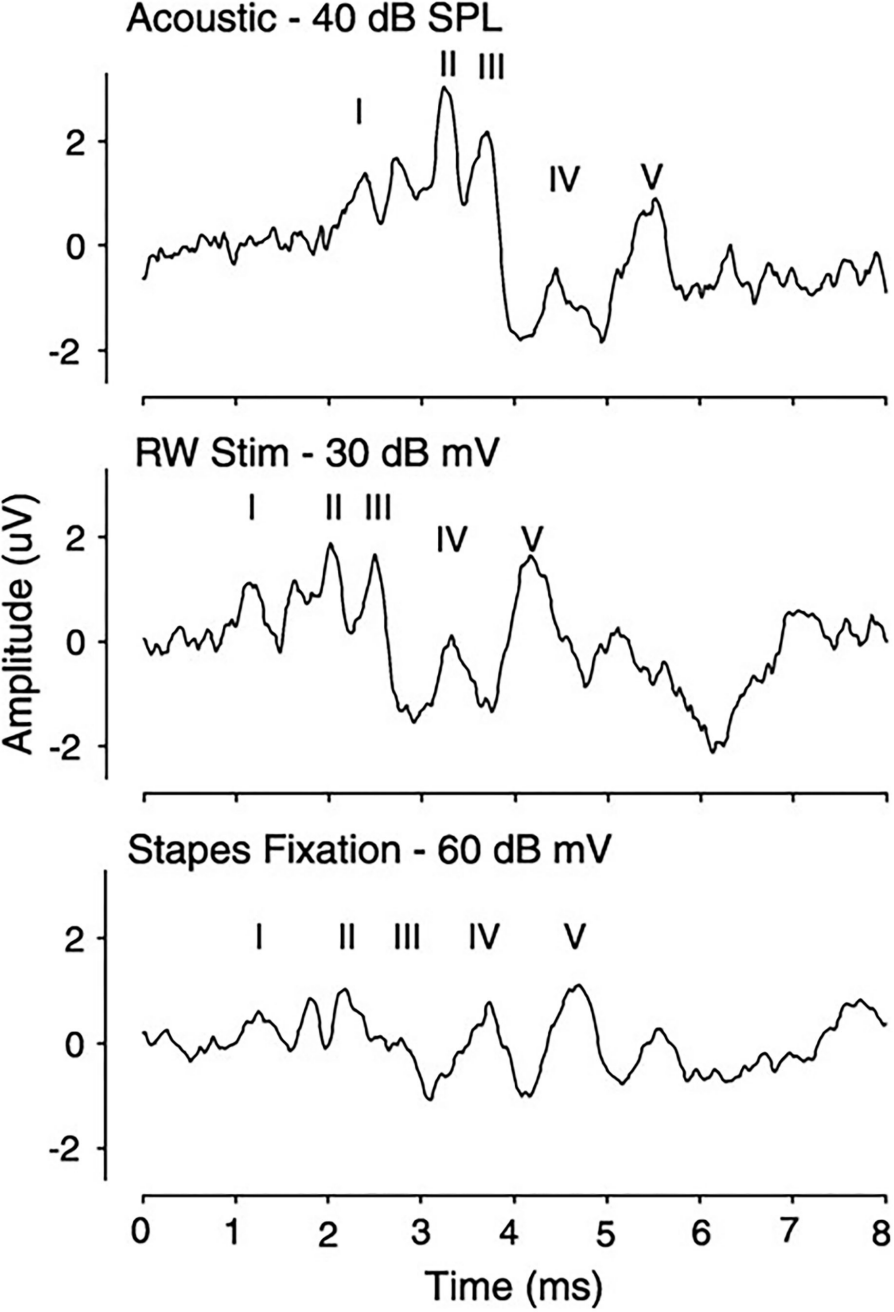
Auditory brainstem response in a subject using a 4-KHz tone-pip stimulation for acoustic, RW, and WE with SF conditions. SF, stapes fixation.

Compound action potential (CAP) measurements, which represent synchronous firing of auditory nerve fibers, are similar in morphology, as shown in Figure 6, regardless of stimulation condition and amplitude (N1-P1) decreased and N1 latency increased with decreasing stimulation intensity. A decreased latency was noted with RW stimulation compared to acoustical, related to the time required for the conducted acoustic signal to reach the cochlea. CAP thresholds were similar in acoustic and RW stimulation but significantly increased with SF, which was increasing prominent at higher frequencies. Recorded ABR responses are similar in morphology for all three stimulus conditions, as shown in Figure 7. The authors (38) concluded that CM, CAP, and ABR findings in Oval Window (OW) mechanical and acoustical stimulations resulted in similar transduction at the hair cell, auditory nerve, and brainstem levels. The presence of SF merely attenuated the response at all levels. Similar results in SF between RW stimulation and a third window vibroplasty with the piston entrance directly into the scala tympani (39) and the lateral semicircular canal (40). Similar results were seen in stimulating the inner ear acoustically in the presence of a simulated middle ear effusion, when compared to direct RW stimulation (41).

A more in-depth look at the ABR in a live Guinea pig model comparing responses to piezoelectric transducer RW stimulation with that of an acoustic stimulus in the external auditory canal showed significant differences in amplitudes and latencies of the ABR to the two stimulations (42). At comparable stapes velocity stimulations, the acoustic ABR had shorter latencies and larger amplitudes than that generated from the RW stimulation. The authors concluded that the differences noted in ABR recordings reflected differences in output functions of the cochlea in forward and reverse stimulations. Zhang et al. (43) using a finite element analysis showed that results in response to reverse stimulation were changed with the material properties of the stapes themselves, depending on whether acoustic or RW stimulation (43). With SF, the pressure difference across the cochlear partition is smaller with RW than sound stimulation.

## Cadaveric Human Temporal Bone Studies

Coupling of the prostheses has been speculated as a cause of the failure by many. Mancheno et al. (44) stressed the variability of the oval and RW anatomy and its implications in obtaining “optimal removal” of the RW niche to better visualize the limits of the membrane (44). Other authors evaluated the coupling of the RW in various conditions of direct placement, interposed fascia, and overall coverage with fat, fascia, or cartilage (45). Using laser Doppler vibrometry, the use of interposed or covering tissue improved stapes vibrometry responses over that of direct coupling, but each of the tissue techniques did not differ in the magnitude of response.

In more extensive studies of the multiple variables of RW exposure and transducer tip diameter in stimulating human cadaveric temporal bones (46), stapes velocity transfer functions were measured in response to acoustic stimulation of the ear and micromechanical stimulation of the RW. Two sizes of ball tips stimulator, 0.5 and 1.0 mm, were assessed in both the normal and drilled-out RW niche. Their studies indicated that the RW membrane could be successfully stimulated for hearing output without drilling the niche. However, stimulation was significantly improved in the medium and higher frequencies with the removal of the bony rim of the niche. The investigators overall concluded that drilling the niche enhanced visualization of the full membrane and facilitated the placement of the stimulator.

The two diameter tips resulted in the same stapes velocity outputs in the low-to-medium frequencies. However, the 0.5-mm tip was 5–6 dB better than the 1 mm at high frequencies both before and after drilling the RW niche. The lack of improvement at the low-to-mid frequencies for the 1 mm ball tip was speculated as potentially resulting from interference of the bony dimensions of the RW, measured as 0.92 mm on average compared to 1.0 mm stimulating ball electrode. With the smaller tip, the entire circumference of the RW membrane could be observed.

Backside loading has been stressed as influencing the performance of an FMT in enhancing its vibratory performance and retention of long-term coupling (47). Using a coupling impedance model of the human ear, Xue et al. (48) determined that poor coupling resulted in deterioration of responses across the entire frequency ranges with RW stimulation (48). Liu et al. (49) using a human ear finite-element model comparison of sites of coupling of the RW membrane with that of the umbo, incus body, incus long process, and stapes demonstrated that for an equal force of stimulation applied, the RW produced more efficient cochlear response than equivalent magnitudes of force stimulating the other sites, such as the stapes footplate (49).

Placing FMT perpendicular to the footplate, i.e., the direction of vibration toward the interposed tissue gave the best results in stapes movement (50). Disarticulation of the ossicular chain did not change the stapes velocity measures. Marked drops in stapes velocity occurred in response with the implant on the side over an oval window.

## Mechanisms Inner Ear Pathway Stimulation

Two different pathways for forward and reverse stimulations have been proposed (51), due largely to the differences in compliance of the RW and the footplate of the ossicular chain. In the forward drive, the large magnitude of difference in SV and ST pressures results from the high compliance, i.e., the two window hypotheses (35). In reverse, the high impedance of the middle ear results in similar pressure magnitudes in the SV and ST, and the phase of the pressures across the cochlear partition becomes a much more prominent stimulator. In reverse stimulation, the efficiency of the coupling to the RW and other potential third window effects is not measured, perhaps the vasculature and/or cochlear aqueduct becomes much more of a potential explanation for the difference in driving the system retrogradely.

Nakajima et al. (52) determined that the differential sound pressure measures across the cochlear partition dividing the SV and ST at the cochlear base are a sensitive measure of cochlear input (52). Sound stimulation results in larger pressure in the SV than in the ST. In a follow-up study, equivalent stimulations of the oval and RW above 1 kHz produced similar pressure changes (53). However, at lower frequencies of the stimulation, the pressure drops, and distortion of the response from RW stimulation was not readily adjustable with increasing voltage. Chen et al. (54) studied the movement of the basilar membrane in response to forward and reverse stimulations, monitoring the movement of the incus tip and the basal coil of the basilar membrane with laser Doppler vibrometer with a reflective bead placed directly on the basilar membrane and visualized through a glass-covered fenestration (54). The basilar membrane movement was similar in forward and reverse drives, with characteristic frequencies for both directions between 13 and 14 Hz. The animals manifest a reduction in the displacement ratio of the basilar membrane, attributed by the authors to potential coupling issues of the magnet to the RW membrane and/or potential leakage of inner ear fluid.

## Conclusions

This review has summarized the utility of the vibroplasty operation and its scientific validation. While many points are still controversial in parameters of surgical techniques and actual changes that may be occurring in forward vs. retrograde stimulation, clinical outcomes have been excellent in general in a population with very little else available to improve inner ear stimulation. The size of the FMT is a problem in many situations, approximately equaling the RW membrane in size (44). The interposed tissue becomes important in still being able to direct a stimulus to the RW membrane in cases in which direct application may be compromised by surrounding bone.

Multiple devices developed for ossicular chain stimulation have been modified for direct stimulation of the RW with successful outcomes. However, while being able to drive the inner ear retrogradely with the improvement of sound perception is very helpful to patients, it is not always commercially feasible to produce such devices. Even though results from essentially all the devices over the years have been significant improvement (55), third-party payers have been reluctant to approve any active middle prostheses. Instead, they have treated them as hearing aid and not an implant, the former not being reimbursed by most insurers. These devices have as a result been essentially abandoned by nearly all manufacturers, except for the FMT by the Med-El Corporation. Vibroplasty using the FMT is still being widely performed in many places in the world with success.

Many refinements need to be made to all components of these devices, including implantable microphones and the problems of designing a translating device to be used long term in a biological system. Though successful results by patients with the APHABs (56) would seem to support adoption for wider use, even with the expense of device and surgical procedures.

Failure of obtaining third-party reimbursement throughout the world will continue to prevent future production and advancements. Even though these needed improvements could be made with present day advances in technology, the decision to withdraw or not the market was made by the company, based on marketing feasibility and needed expenditure to perfect the stimulating devices. Today, only the FMT is being manufactured and marketed worldwide, while all others have been removed.

## Author Contributions

HJ primary author in developing concept research and writing. NG and DT advised on concept and aided writing manuscript. All authors contributed to the article and approved the submitted version.

## Conflict of Interest

The authors declare that the research was conducted in the absence of any commercial or financial relationships that could be construed as a potential conflict of interest.

## Publisher's Note

All claims expressed in this article are solely those of the authors and do not necessarily represent those of their affiliated organizations, or those of the publisher, the editors and the reviewers. Any product that may be evaluated in this article, or claim that may be made by its manufacturer, is not guaranteed or endorsed by the publisher.
